# A Case of Iatrogenic Panhypopituitarism: Exploring Psychological Symptoms and Psychiatric Interventions

**DOI:** 10.1192/j.eurpsy.2025.1238

**Published:** 2025-08-26

**Authors:** L. Kozina, L. Murn, J. Sušac

**Affiliations:** 1 University Psychiatric Hospital Vrapče; 2 Psychiatry Department, Clinical Hospital Dubrava, Zagreb, Croatia

## Abstract

**Introduction:**

Panhypopituitarism is a rare and life-threatening endocrine condition characterized by the loss of pituitary hormone function. Literature mentions the very rare occurrence of iatrogenic panhypopituitarism caused by neurosurgical operations, radiotherapy, and pharmacotherapy – long-term use of corticosteroids can suppress the HPA axis, leading to secondary cortisol insufficiency, which, in very rare cases, progresses to global panhypopituitarism. Psychological symptoms are often present but are unfortunately rarely recognized.

**Objectives:**

A case of a patient with psychological symptoms due to panhypopituitarism is presented.

**Methods:**

A case report is presented with a literature review.

**Results:**

A 37-year-old patient, has experienced fatigue, tremors, loss of sexual desire, blood pressure and glucose fluctuations, and impaired temperature and pain sensation. Endocrinological testing revealed insufficiency of all pituitary hormones. Multiple MRI scans of the brain showed a normal appearance of the pituitary gland. It was discovered that he had a severe car accident with a head injury 10 years ago. Since then, he has been taking high doses of corticosteroids on his initiative due to severe spinal pain. It is believed that the condition developed iatrogenically due to corticosteroid medication. His psychological condition significantly worsened after one of the adrenal crises. He describes an experience of “encountering death” during one of the adrenal crises. Furthermore, he describes that after this adrenal crisis, his corticosteroid dosage was significantly increased, resulting in feelings of excessive energy, worse anxiety, insomnia, and irritability. Because of this, he began self-reducing the corticosteroid dose, leading to a “vicious cycle” where he fears another crisis. In complex patients like this one, it is crucial to develop a comprehensive treatment plan and ensure good collaboration with somatic physicians. This patient presents with depressive and anxiety symptoms within the context of an adjustment disorder. Additionally, corticosteroid therapy contributes to emotional instability, and the patient also exhibits symptoms of PTSD due to a near-death experience. Also, the patient’s complex psychodynamic profile presents significant challenges to treatment. The therapeutic goals for this patient are: mood stabilization and anxiety reduction, sleep regulation, and breaking the “vicious cycle”. CBT is the treatment of choice for addressing the patient’s anxiety, fear of adrenal crisis, and self-reduction of corticosteroid doses. The patient’s complex psychodynamics and high cognitive functioning make him an excellent candidate for long-term psychodynamic psychotherapy.

**Conclusions:**

The psychiatrist plays a crucial role in treating such complex patients, and close collaboration with somatic physicians, along with an adequate and thorough therapeutic treatment plan, is necessary.

**Disclosure of Interest:**

None Declared

